# A Selective α7 Nicotinic Acetylcholine Receptor Agonist, PNU-282987, Attenuates ILC2s Activation and *Alternaria*-Induced Airway Inflammation

**DOI:** 10.3389/fimmu.2020.598165

**Published:** 2021-02-01

**Authors:** Fang Yuan, Lili Jiang, Qianyang Li, Leon Sokulsky, Yuanyuan Wanyan, Lingli Wang, Xiaojie Liu, Lujia Zhou, Hock L. Tay, Guojun Zhang, Ming Yang, Fuguang Li

**Affiliations:** ^1^ Academy of Medical Sciences and Department of Immunology, College of Basic Medical Sciences, Zhengzhou University, Zhengzhou, China; ^2^ Department of Medical Laboratory, The Second Affiliated Hospital of Zhengzhou University, Zhengzhou, China; ^3^ Priority Research Centre for Healthy Lungs, Faculty of Health and Hunter Medical Research Institute, School of Biomedical Sciences and Pharmacy, University of Newcastle, Callaghan, NSW, Australia; ^4^ Department of Respiratory and Critical Care Medicine, The First Affiliated Hospital of Zhengzhou University, Zhengzhou, China

**Keywords:** type 2 innate lymphoid cells, airway, inflammation, nicotinic acetylcholine receptor, Alternaria

## Abstract

**Background:**

The anti-inflammatory effect of an α7nAChR agonist, PNU-282987, has previously been explored in the context of inflammatory disease. However, the effects of PNU-282987 on type 2 innate lymphoid cells (ILC2s)-mediated allergic airway inflammation has not yet been established.

**Aims:**

To determine the effects of PNU-282987 on the function of ILC2s in the context of IL-33– or *Alternaria Alternata* (AA)– induced airway inflammation.

**Methods:**

PNU-282987 was administered to mice that received recombinant IL-33 or AA intranasal challenges. Lung histological analysis and flow cytometry were performed to determine airway inflammation and the infiltration and activation of ILC2s. The previously published α7nAChR agonist GTS-21 was employed as a comparable reagent. ILC2s were isolated from murine lung tissue and cultured *in vitro* in the presence of IL-33, IL-2, and IL-7 with/without either PNU-282987 or GTS-21. The expression of the transcription factors GATA3, IKK, and NF-κB were also determined.

**Results:**

PNU-282987 and GTS-21 significantly reduced goblet cell hyperplasia in the airway, eosinophil infiltration, and ILC2s numbers in BALF, following IL-33 or AA challenge. *In vitro* IL-33 stimulation of isolated lung ILC2s showed a reduction of GATA3 and Ki67 in response to PNU-282987 or GTS-21 treatments. There was a significant reduction in IKK and NF-κB phosphorylation in the PNU-282987–treated group when compared to the GTS-21–treated ILC2s.

**Conclusion:**

PNU-282987 inhibits ILC2-associated airway inflammation, where its effects were comparable to that of GTS-21.

## Introduction

Asthma is an immune disorder of the lungs associated with airway inflammation, mucus secretion, and airway hyperresponsiveness (AHR), and is associated with the type 2 cytokines, interleukin (IL)-4, IL-5, and IL-13 (1, 2). While CD4^+^ T helper 2 (Th2) cells play an important role in the development of allergic cascades, innate immune cells including macrophages, eosinophils, and type 2 innate lymphoid cells (ILC2s) critically contribute to the pathogenesis of the disease (3). Classically, Th2 cells were regarded as the central cell involved in asthma, as they were believed to be the only cellular source of type 2 cytokines. However, the discovery of the ILC2s highlighted a new class of cells that can induce inflammatory cascades independent of T and B cells in response to epithelial alarmin factors, such as IL-25 and IL-33 (4, 5). Upon exposure to these factors, ILC2s secrete Th2 cytokines including IL-4, IL-5, and IL-13 through GATA3 transcription (6–9). Indeed, Rag2^−/−^ mice that lack T cells and B cells presented with pathological features of allergic airway disease due to the presence of ILC2s (10). Furthermore, mouse models of asthma have revealed that the genetic depletion of IL-33 results in a marked reduction of eosinophilic inflammation and mucus secretion following aerosol allergen exposure, indicating the key role of IL-33 in the pathogenesis of asthma (11). IL-33 initiates inflammation by activating ST2/IL-1RAcP, which in turn recruits IRAKs and TRAF6 for the activation of IKK, NF-κB, and MAPK signaling pathways (10). In Rag2^−/−^Il2rg^−/−^ mice (lacking T cells, B cells, and ILCs), the levels of eosinophilic inflammation and the secretion of mucus decreased significantly, highlighting the importance of the IL-33/ILC2s axis in the development of asthma (12).

Importantly, Tracey et al. identified a cholinergic anti-inflammatory pathway (CAP) that is mediated by the vagus nerve, a major neurological regulator of organ function throughout the body (12, 13). CAP controls inflammation through the release of the neurotransmitter acetylcholine (12, 13). Acetylcholine can also stimulate the α7 nicotinic acetylcholine receptor (α7nAChR) that is expressed on macrophages, T cells, and B cells (14–16). Animal models of diabetes, sepsis, cystic fibrosis, ulcerative colitis, and arthritis in α7nAChR-deficient mice have revealed that this receptor downregulates the function of macrophages and lymphocytes, and attenuates the development of pathology (17–21). Interestingly, recent observation has found that ILC2s expression of α7nAChR is significantly higher than that on macrophages and other lymphocytes (22).

GTS-21 is recognized as an α7nAChR agonist that also binds to α4β2nAChR with high affinity, where the binding power of the latter receptor is 100 times higher compared to the former. α4β2nAChR is predominantly expressed in the central nervous system and regulates psychological activities including mood, memory, and learning (23). Previously, Lauriane Galle-Treger et al. demonstrated that GTS-21 has a significant inhibitory effect on ILC2s-mediated airway inflammation, suggesting GST-21 could be exploited therapeutically to suppress ILC2s-associated inflammatory disorders (22). However, its action on α4β2nAChR may yield unwanted side-effect as the aberrant activation of this receptor leads to anxiety, downregulation of D3 dopamine receptor and interference of growth hormone release (24–26). Interestingly, the compound PNU-282987 has a higher affinity to α7nAChR with little or no effect on the α1, β1, γδ, and α3β4 variants, and monoamine, muscarinic, glutamate, and GABA receptor (27). Moreover, PNU-282987 has been demonstrated to reduce acute lung injury by altering macrophage proliferation in mice (28). Therefore, we plan to explore the effects of PNU-282987 on ILC2s and to compare the difference between PNU-282987 and GTS-21 on ILC2s using both *in vivo* models and *in vitro* stimulation of isolated ILC2s from mouse lung.

## Materials and Methods

### Mice

6 to 8 week old C57BL/6J female mice were purchased from Beijing Vitonlihua Company [license No. SCXK (Beijing) 2012/0001] and were raised at the SPF animal housing facility, Zhengzhou University. The experiment was approved and permitted by the Animal Ethics Committee of Zhengzhou University (Approval Number: ZZURIB20180120).

### Murine Model

Mice were intranasally (i.n.) administered with recombinant mouse IL-33 (0.5 µg/dose, R&D, California, USA) in mice intraperitoneally (i.p.) administered with PNU-282987 (20 mg/kg, Abcam, California, USA) or GTS-21 (20 mg/kg, Abcam, California, USA) over three consecutive days (22). For *Alternaria* experiments, mice were i.n. administered with AA (100 µg/dose, Greer Labs, Lenoir, North Carolina, USA) in the presence or absence of PNU-282987 or GTS-21 on four consecutive days. Mice were sacrificed on the second day after the last challenge.

### Collection of Bronchoalveolar Lavage Fluid (BALF) Cells and Lung Histology

Mouse lung was lavaged three times with 0.8 ml of ice-cold PBS for the collection of BALF cells. Supernatants were collected for ELISA. Red blood cells were then removed by using hypotonic red blood cell lysis buffer and BALF was then centrifuged to collect cellular infiltrate. Total cell numbers were quantified using a hemocytometer. In some experiments, lung tissues were digested with Liberase™ and DNase (Roche, Basel, Switzerland) for single-cell suspensions. BALF and lung cells were analyzed *via* flow cytometry.

In additional experiments, lung tissues were stained with hematoxylin and eosin (for histopathology) or periodic acid-Schiff (for mucus-secreting cells). Sections were then stained with chromotrope-hematoxylin or periodic acid-Schiff (PAS). Scorings for histopathology (inflammatory infiltrates) and PAS (mucus-producing cells) were performed according to a set of morphological criteria as previously described (29).

### Flow Cytometry

Purified rat anti-mouse CD16/CD32 (553141), PE-conjugated hamster anti-mouse KLRG1 (561621), PerCP-Cy™5.5-conjugated hamster anti-mouse KLRG1 (563595), FITC-conjugated rat anti-mouse Ly-6A/E (557405), PE-conjugated rat anti-mouse Siglec-F (552126) and PE-conjugated rat anti-mouse/anti-human IL-5 (562019) were purchased from BD Biosciences. PE-conjugated anti-IL-13 monoclonal antibody (eBio 13A) was purchased from Invitrogen (San Diego, California, USA). PE-conjugated anti-mouse phospho-IKKα/β (Ser 176/180) and Alexa Fluor 488-conjugated anti-mouse phospho-NF-κB p65 (Ser 536) (93H1) rabbit mAbs were purchased from Cell Signaling Technology (Danvers, Massachusetts, USA). Lung ILC2s was defined as lack of classical lineage markers (CD5, CD45R, Anti-Gr-1, CD11b, 7-4, and Ter119), KLRG1^+,^ and Sca-1^+^ populations. Lineage marker negative cells were enriched by both density gradient centrifugation and magnetic beads isolation and purified by flow cytometry for the collection of ILC2s.

Intracellular staining was performed with BD Fixation and Permeabilization Solution (BD Biosciences, San Jose, California, USA) according to the manufacturer’s instructions. Cell stimulants and protein transport inhibitors were added before staining. The analysis of IKK-P and NF-κB p65 was carried out per the manufacturer’s instructions. FACSCanto II Flow (BD Biosciences, San Jose, California, USA) and MoFlo XDP cell sorter (Beckman coulter, Brea, California, United States) were employed for flow cytometry and cell sorting. Data were analyzed with software FlowJo version 10.0 (Franklin Lakes, New Jersey, United States). The gating strategy was shown in **Figures S1** and **S2**.

### *In Vitro* Stimulation of ILC2s

ILC2s were isolated and cultured in 96-well plates with a volume of 100 µl per well in RPMI-1640 medium (BD Biosciences, San Jose, CA). The cells were plated at 1.5 × 10^4^ cells per well and stimulated with IL-33 (50 ng/ml, BD Biosciences, San Jose, California, USA), IL-2 (20 ng/mL, BD Biosciences, San Jose, California, USA), and IL-7 (20 ng/mL, BD Biosciences, San Jose, California, USA) in the presence or absence of PNU-282987 (20 μM) or GTS-21 (20 μM) for 24 or 72 h.

### Proliferation Assay Method

0.4% trypan blue staining solution was absorbed with a dropper and added to the cell suspension at 1:1. The staining solution was dropped gently from the edge of the counting board. Cell count was performed 1 min after staining under light microscopy. The concentration of cell suspension and the ratio of survival to dead cells were then calculated.

### Quantitative PCR

Total RNA was extracted and the levels of IL-5, IL-13, GATA3, and IL-33 transcripts were quantitated at the mRNA level by quantitative real-time RT-PCR with Applied Biosystems QuantStudio™ 5 system (Applied Biosystems, Carlsbad, California, USA) following the manufacturer’s protocol. The primers were as follows: IL-5 (forward, 5′-TGAGACGATGAGGCTTCCTG-3′ and reverse, 5′-CCACACTTCTCTTTTTGGCGG-3′) (30), IL-13 (forward, 5′-CCCTCAGCCATGAAATAACT-3′ and reverse, 5′-GCGTAACAGGCCATTCTTCC-3′) (30), GATA3 (forward, 5′-CGAGATGGTACCGGGCACTA-3′ and reverse, 5′-GACAGTTCGCGCAGGATGT-3′) (31), IL-33 (forward, 5′-ACTATGAGTCTCCCTGTCCTG-3′ and reverse, 5′-ACGTCACCCCTTTGAAGC-3′) (32).

### ELISA

IL-5 and IL-13 from BALF and *in vitro* culture supernatants were detected according to the manufacturer’s instructions (MultiSciences Biotech, Hangzhou, Zhejiang, China). Data detection and analysis were performed on a microplate reader (Molecular Devices, San Jose, California, United States).

### Statistical Analysis

SPSS21.0 software was used for statistical analysis. One-way ANOVA was used to compare multiple samples. The results were expressed as the mean ± standard error of the mean (SEM). When *P* < 0.05, the difference between samples is considered statistically significant.

## Results

### PNU-282987 and GTS-21 Inhibit IL-33–Induced Airway Inflammation

**Figure 1:**
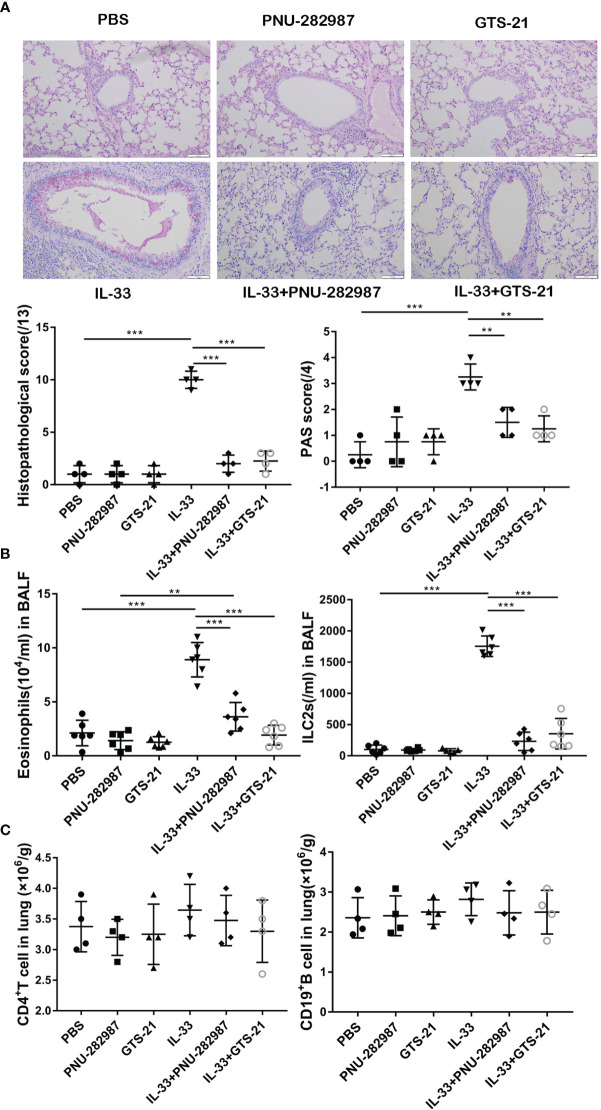
Compared with GTS-21, PNU-282987 has the same inhibitory effect on ILC2-mediated airway inflammation. C57BL/6J mice intranasally challenged with recombinant mouse IL-33 or PBS and also received PNU-282987/GTS-21 or PBS on days 1 to 3. Assessment of lung tissue and analysis of bronchoalveolar lavage fluid (BALF) **(A)** Periodic acid-Schiff reagent lung section (×200, Scale bars at 100 μm). Scorings for histopathology and PAS were assessed. **(B)** Total number of eosinophils and ILC2s in BALF. **(C)** Total number of CD4^+^ T cells and CD19^+^ B cells determined by flow cytometry in lung. Data are representative of at least four independent experiments and presented as means ± s.e.m (n = 4 − 6; **P < 0.01; ***P < 0.001).

Both IL-33 and ILC2s are critically involved in the early stages of asthma exacerbation (33). Firstly, by using recombinant IL-33 to induce lung inflammation *in vivo*, we explored the effects of PNU-282987 and GTS-21 on IL-33–mediated airway inflammation. PAS and HE staining of lung tissue revealed a significant increase of goblet cells in the airway epithelium of the IL-33–exposed group when compared to the PBS group (**Figure 1A**). Treatments with PNU-282987 and GTS-21 significantly attenuated goblet cell hyperplasia and mucus production in airways, and reduced the histopathological scores following IL-33 i.n. inoculation (**Figure 1A**). We also examined the infiltration of eosinophils and ILC2s in BALF and showed that both compounds drastically decreased the migration of both cells (**Figure 1B**). Furthermore, both agonists showed similar responses in the suppression of IL-33–induced airway inflammation and mucus hypersecretion. The populations of CD4^+^ T cells and CD19^+^ B cells in the three groups were unchanged between the IL-33–treated groups (**Figure 1C**), suggesting that the inhibitory action of PNU-282987 and GTS-21 is through their action on ILC2s.

### PNU-282987 and GTS-21 Attenuate the Production of IL-5 and IL-13 Secreted by ILC2s *In Vivo*

**Figure 2:**
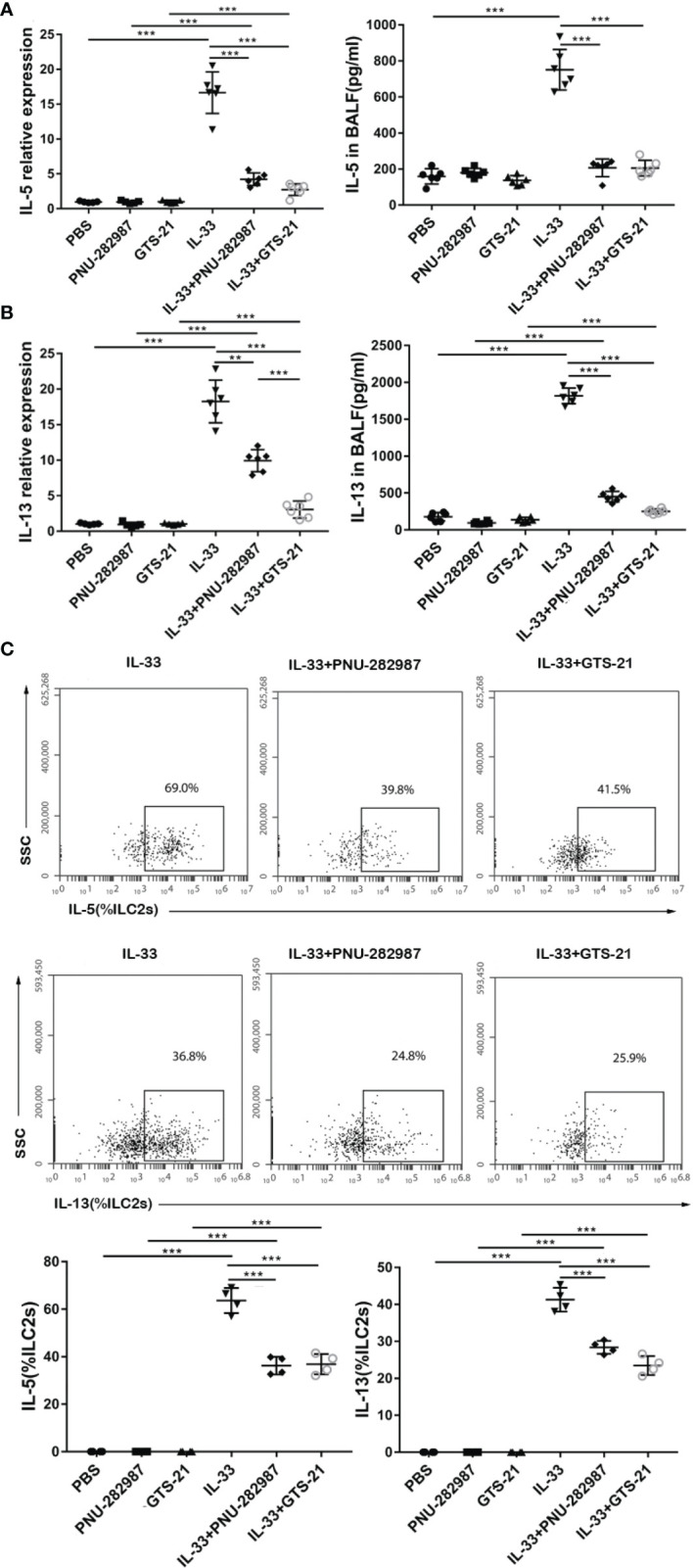
PNU-282987 and GTS-21 attenuate the production of type 2 cytokines secreted by ILC2s *in vivo*. Levels of RNA transcript in lung and protein in BALF of **(A)** IL-5 and **(B)** IL-13 were measured by qPCR and ELISA respectively. **(C)** Percentage of IL-5^+^ILC2s and IL-13^+^ILC2s were determined by flow cytometry. Data are representative of at least four independent experiments and presented as means ± s.e.m (n = 4 - 6; **P < 0.01; ***P < 0.001).

Next, we determined whether PNU-282987 and GTS-21 inhibit the secretion of IL-5 and IL-13 by ILC2s. RNA extracted from mouse lung tissue was used to determine the transcription levels of IL-5 and IL-13, and the protein levels of IL-5 and IL-13 in BALF were also examined. Levels of IL-5 and IL-13 transcripts in the IL-33 treatment group were significantly higher than in the control PBS group. Treatments with PNU-282987 or GTS-21 significantly decreased the levels of IL-5 and IL-13 transcripts in lung and protein in BALF following IL-33 treatment (**Figures 2A, B**). Although the levels of IL-13 mRNA in the IL-33/GTS-21 group were lower than that in IL-33/PNU-282987 group, there was no difference in the BALF protein level of IL-13 between the two groups (**Figure 2B**). Furthermore, there was no significant difference between PNU-282987 and GTS-21 in inhibition of IL-5^+^ILC2s and IL-13^+^ILC2s (**Figure 2C**). No IL-5– or IL-13–secreting ILC2s were identified in PBS, PNU-282987, or GTS-21 treatment groups.

### PNU-282987 and GTS-21 Abolish AA-Mediated Airway Inflammation

**Figure 3:**
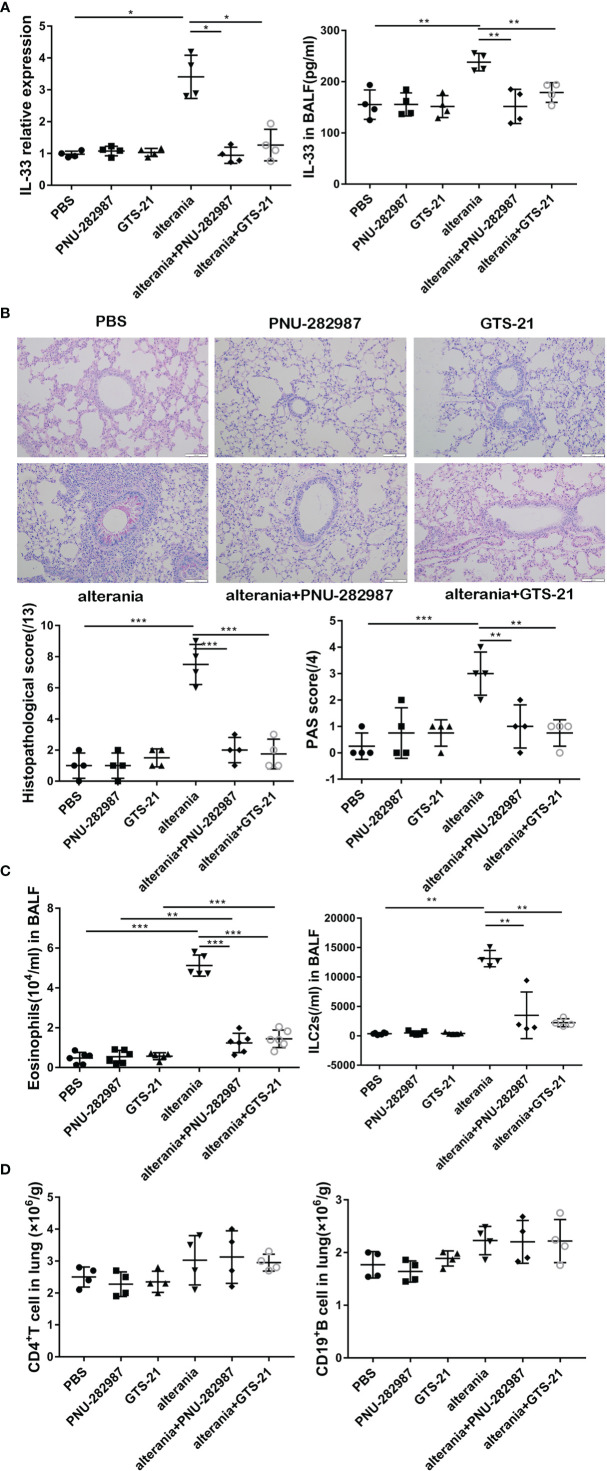
PNU-282987 and GTS-21 inhibit Alternaria-mediated airway inflammation. C57/BL6J mice intranasally received an extract of Alternaria (AA), AA with or without PNU-282987/GTS-21 on days 1 to 4. **(A)** levels of IL-33 RNA in lung and protein in BALF were assessed by qPCR and ELISA respectively. **(B)** Lung tissues were stained by PAS staining. Scoring for histopathology and PAS was assessed by light microscopy (×200, Scale bars at 100 μm). **(C)** Levels of eosinophils and ILC2s in BALF, **(D)** CD4^+^ T cells and CD19^+^ B cells in lung were determined by flow cytometry. Data are representative of at least four independent experiments and presented as means ± s.e.m (n = 4 - 5; *P < 0.05; **P < 0.01; ***P < 0.001).

We then determined the anti-inflammatory effects of PNU-282987 on ILC2-regulated airway inflammation and compared its function to that of GTS-21 with AA- challenged animals (22). Our experiments confirmed that the level of IL-33 in the AA treatment group was significantly higher than that in the PBS group (**Figure 3A**). After the administration with PNU-282987 and GTS-21, the levels of RNA in lung and protein in BALF of IL-33 were significantly decreased when compared to the AA alone group. Furthermore, treatments with PNU-282987 or GTS-21 attenuated the AA-induced airway inflammation when we examined the histopathological changes of lung tissue (**Figure 3B**). The levels of eosinophil and ILC2s in the BALF of the AA/PNU-282987 and AA/GTS-21 groups were significantly lower than those of AA only group, and there was no difference between the two former groups (**Figure 3C**). There was no difference in the levels of CD4^+^ T cells and CD19^+^ B cells in lung between AA, AA/PNU-282987, and AA/GTS-21 groups (**Figure 3D**). These results suggest that PNU-282987 also inhibits the ILC2-mediated airway inflammation caused by the AA.

### PNU-282987 and GTS-21 Inhibit the Production of IL-5 and IL-13 in the Lung of AA-Challenged Mice

**Figure 4:**
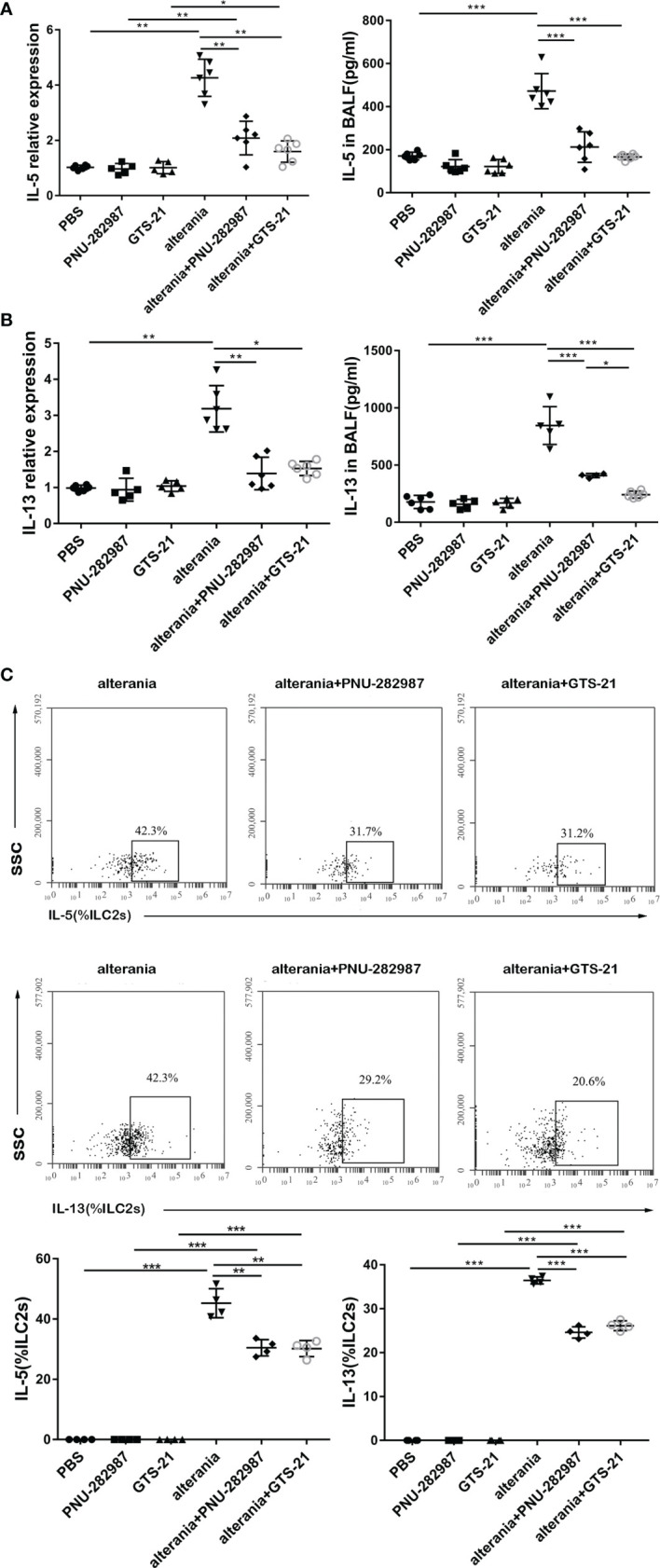
PNU-282987 and GTS-21 inhibit the function of ILC2s in Alternaria-mediated Airway inflammation. Levels of RNA transcript in lung and protein in BALF of **(A)** IL-5 and **(B)** IL-13 were measured by qPCR and ELISA respectively. **(C)** Percentage of IL-5^+^ILC2s and IL-13^+^ILC2s was determined by flow cytometry. Data are representative of at least four independent experiments and presented as means ± s.e.m (n = 4 - 6; *P < 0.05; **P < 0.01; ***P < 0.001).

Similar to what occurred in the IL-33 model, PNU-282987 and GTS-21 inhibited the production of IL-5 and IL-13 by ILC2s isolated from mice that were exposed to AA, both at transcription and protein levels (**Figures 4A, B**). Furthermore, intracellular staining revealed that the levels of IL-5– and IL-13–producing ILC2s in the AA/PNU-282987 and AA/GTS-21 groups were profoundly reduced compared to the AA alone group (**Figure 4C**). There was no difference between PNU-282987 and GTS-21 in the inhibition of the IL-5– and IL-13–producing function of ILC2s.

### PNU-282987 and GTS-21 Inhibited the Proliferation and Functional Activation of ILC2s *In Vitro*

**Figure 5:**
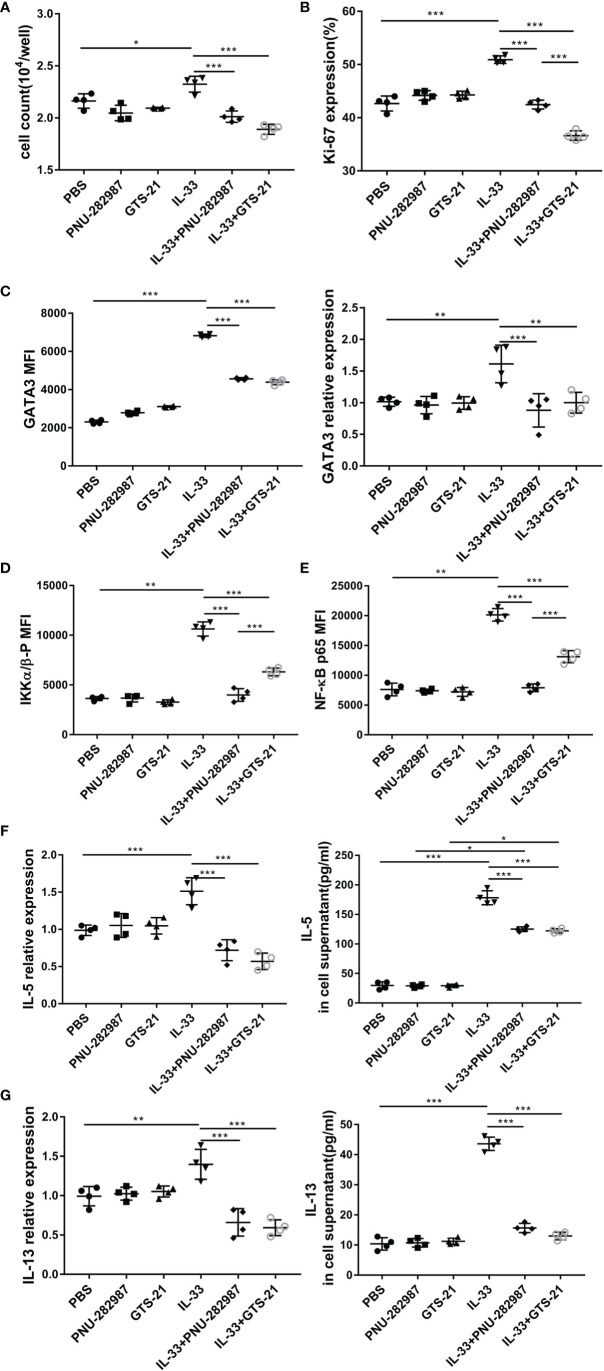
PNU-282987 and GTS-21 inhibit the proliferation and functional activation of ILC2s *in vitro*. Lin^-^ cells from the lung of C57/BL6J female mice were first separated by magnetic beads, and ILC2s were further isolated by FACS. **(A)** Cell count of ILC2s and **(B)** Percentage of Ki67^+^cells in isolated lung ILC2s, stimulated with IL-33 in the absence and presence of PNU-282987/GTS-21 for 72 h. **(C)** Mean fluorescence intensity and the RNA levels of GATA3 in isolated lung ILC2s were analyzed by flow cytometry and qPCR. MFI of phosphorylated **(D)** IKKα/β and **(E)** NF-κB p65 with or without PNU-282987/GTS-21 for 24 h. RNA and protein levels of **(F)** IL-5 and **(G)** IL-13 from cultured ILC2s were measured by qPCR and ELISA. Data are representative of at least four independent experiments and presented as means ± s.e.m (n = 4; *P < 0.05; **P < 0.01; ***P < 0.001).

ILC2s from mouse lung tissue were purified by flow cytometry and cultured with or without PNU-282987 or GTS-21 in the presence of IL-2+IL-7 and/or IL-33. After 72 h of culture, the cell numbers were determined for each group, where it was found that that PNU-282987 or GTS-21 administration was associated with a significantly decreased proliferation of ILC2s despite IL-33 exposure (**Figure 5A**). Per the above results, Ki67, a nuclear non-histone that represents cell proliferation, was significantly decreased in the PNU-282987 and GTS-21 groups following IL-33 exposure (**Figure 5B**). The transcription factor GATA3, which is essential for the growth and development of ILC2s, was also inhibited in the PNU-282987 and GTS-21 groups (**Figure 5C**) (34). Data plots for Ki67 and GATA3 of ILC2s were shown in **Figures S3** and **S4**. PNU-282987 and GTS-21 had comparable inhibitory effects on both Ki67 and GATA3.

IKK and NF-κB p65 levels were evaluated to determine the effects of PNU-282987 and GTS-21 on the proliferation and activation of ILC2s (35). Our results showed that both PNU-282987 and GTS-21 could inhibit the phosphorylation of IKK and NF-κB p65, however the inhibitory effect of PNU-282987 on the phosphorylation of IKK and NF-κB p65 was significantly higher than that of GTS-21 24 h after treatments (**Figures 5D, E**). Seventy-two hours after stimulation, there was no detection of the phosphorylation of these two transcriptional regulators among all groups (data not shown). NF-κB pathway critically regulates the expression of IL-6, BCL-6, c-FLIP, and ST2 that play important role in cell proliferation and inflammation (36). Moreover, IL-33 significantly increased ST2 and c-FLIP expression, which were suppressed by the addition of PNU-282987 and GTS-21 (**Figure S5**). Interestingly, both antagonists had no effect on IL-33–induced higher expression of IL-6 and BCL-6 (**Figure S5**). Cell supernatants were collected, and the levels of IL-5 and IL-13 were assayed by ELISA after 72 h of culture. The results showed that PNU-282987 and GTS-21 had the same effect on inhibiting the secretion of IL-5 and IL-13 by ILC2s (**Figures 5F, G**).

## Discussion

Fungal allergens are a common household cause of asthma, where fungal proteases are potent initiators of allergic inflammatory cascades (37). Such inflammatory cascades induce the release of alarmin factors (e.g. IL-33), which critically drive the activation of ILC2s and increase secretions of type 2 cytokines. The recent discovery of vagus nerve neuro-regulation of ILC2s through CAP and the α7nAChR has highlighted a novel pathway that could be exploited to attenuate asthma, which has previously been demonstrated with the α7nAChR agonist GTS-21 (22). Here, we demonstrated that the agonist PNU-282987 not only exhibits the same inhibitory effects of GTS-21 but also acts as a potent suppresser of IKK and NF-κB activity in ILC2 cells.

We have firstly employed both recombinant IL-33 and AA to challenge mice for the induction of airway inflammation. Our results showed enhanced infiltration of inflammatory cells and increased levels of proinflammatory cytokines including IL-1β, IL-6, and TNF-α (**Figures S6** and **S7**). Furthermore, we have demonstrated that PNU-282987 reduces eosinophil and ILC2 numbers in BALF and lung tissue and decreases goblet cell hyperplasia in the airway. Although CD4^+^ Th2 cells are also the major cellular source of IL-5 and IL-13, we did not observe any changes of their infiltration. Our IL-33 and AA models are both short model, therefore it is unlikely that CD4^+^ antigen specific Th2 cells can differentiate within such narrow time window, highlighting the importance of ILC2s in the disease. In both IL-33- and AA- exposed mice, PNU-282987 and GTS-21 not only abated the proliferation of ILC2s but also inhibited the ability of ILC2s to secrete IL-5 and IL-13 *in vivo*. Furthermore, the inhibition effects of PNU-282987 and GTS-21 on IL-33 protein in BALF are potentially linked to the reduced level of IL-33 protein release from the cells. These data suggest that both PNU-282987 and GTS-21 reduce type 2 cytokines by acting on ILC2s, and that both agonists may directly regulate upstream effect by regulating pre-synthesized IL-33 protein release. Notably, these two cytokines are involved in eosinophil recruitment, AHR, and mucus production (38). While the effectiveness of PNU-282987 and GTS-21 in inhibiting the function of ILC2s was comparable to each other, the former one had a stronger inhibitory effect on IKK and NF-κB, which play a key role in the growth and development of ILC2s (35). This provides stronger evidence for the future study of a unique α7nAChR/IKK/NF-κB focused pathway. Furthermore, the suppression of two NF-κB associated molecules, c-FLIP and ST2, supports the key roles of NF-κB in the differentiation of ILC2s. Through these experiments, we have demonstrated that in addition to GTS-21, PNU-282987 inhibits airway inflammation associated with asthma by interfering with the proliferation and function of ILC2s.

To further explore through what transcription pathways PNU-282987 and GTS-21 enact in ILC2s, flow cytometry was used to assess the expression of the cell proliferation marker, Ki67, and the transcription factors, GATA3, on ILC2s, which are essential for cell proliferation and the production of IL-5 and IL-13. As we expected, the expressions of Ki67 and GATA3 were down-regulated in IL-33/PNU-282987 group when compared to the IL-33 group. This was not surprising as many α7nAChRs-mediated pathways are involved in the process of inhibiting inflammation. This includes the JAK2-STAT3 signaling pathway, α7nAChR/IKK/NF-κB signaling pathway, and α7nAChR/MyD88/IKK/NF-κB pathway (39, 40). STAT3 activated by α7nAChR is a negative regulator of the inflammatory response, and in α7nAChR/IKK/NF-κB signaling axis, α7nAChR further inhibits the nuclear translocation of NF-κB by inhibiting the phosphorylation of upstream signal IKK (41). As Dowling et al. found that nicotine can inhibit the NF- κB signal pathway by binding to α7nAChR, this result supports the notion that the anti-inflammatory mechanism of α7nAChR may be related to IKK and NF- κB transcriptional regulators (42, 43).

We also determined that α7nAChR interacts with I-κB kinase (IKK) and NF-κB as I-κB kinase (IKK) induces NF-κB nuclear activation. After ILC2s were cultured for 24 h *in vitro*, we found that PNU-282987 inhibited the expression of IKK and NF-κB p65 at a significantly higher level when compared with GTS-21. For the different inhibitory effects of PNU-282987 and GTS-21 on IKK and NF-κB, we hypothesized that this observation may be related to the binding ability of GTS-21 to other nicotine receptors, which may interfere with the inhibitory effects of this agonist on α7nAChR activities (44, 45).

Acetylcholine is a major parasympathetic neurotransmitter that has previously been shown to effectively inhibit the release of inflammatory cytokines, such as TNFα from peripheral macrophages stimulated by LPS *in vitro* (13). Stimulation of efferent vagus nerve can inhibit systemic inflammation, as demonstrated in a study that the level of TNFα in serum and liver decreased significantly in Lewis rats with endotoxemia after cervical vagotomy by electric stimulation (13). Furthermore, the release of norepinephrine by the vagus nerve stimulates memory T cells to secrete acetylcholine for the negative regulation of inflammation on α7nAChR-positive cells (46, 47). Our finding that ILC2-driven airway inflammation is negatively regulated by α7nAChR is supported by the observation that ILC2 activity is reduced by memory T-cell release of acetylcholine (48). Interestingly, it is also known that ILC2s express β_2_-adrenergic receptors (that respond to norepinephrine), which when activated will agonistically suppress ILC2 proliferation and secretion of type 2 cytokines (49). While this study only focused on exploiting α7nAChR-agonism to regulate ILC2 inflammation, the effects of norepinephrine on the inflammatory cascades explored above should be further investigated to determine if any overlap exists between the two neuro-inhibitory pathways.

Based on our *in vivo* data, the viability of ILC2s in culture was inhibited by PNU-282987. Except for IKK and NF-κB, there was no significant difference in the proliferation of ILC2s and the secretion of IL-5 and IL-13 between PNU-282987 and GTS-21 *in vitro.* Although this mechanism needs to be explored further, it is clear that our results show that PNU-282987 is effective in the treatment of ILC2-mediated airway inflammation induced by IL-33 and AA. The importance of the cholinergic anti-inflammatory pathway and α7nAChR as a pharmacological target for the treatment of inflammatory diseases needs further investigation. More importantly, the inhibitory effect of PNU-282987 on IKK and NF-κB should warrant further attention and it is worthy of future exploration.

## Data Availability Statement

The raw data supporting the conclusions of this article will be made available by the authors, without undue reservation.

## Ethics Statement

The animal study was reviewed and approved by Animal Ethics Committee of Zhengzhou University.

## Author Contributions

Performed the experiments: FY, LJ, QL, YW, WL, HT. Analyzed and interpreted the data: FY, QL, XL, LZ, FL, MY. Conceived and designed the experiments: FL, MY. Wrote and edited the paper: FY, LS, GZ, FL, MY. All authors contributed to the article and approved the submitted version.

## Funding

This work was supported by the Henan Provincial Higher Education Key Research Project Grant (18A310030), China National Natural Science Foundation Project Grant (#81970030).

## Conflict of Interest

The authors declare that the research was conducted in the absence of any commercial or financial relationships that could be construed as a potential conflict of interest.
