# Supplementary figures and images for: A Selective α7 Nicotinic Acetylcholine Receptor Agonist, PNU-282987, Attenuates ILC2s Activation and Alternaria-Induced Airway Inflammation
Source: Front Immunol. 2021 Feb 1;11:598165. doi: 10.3389/fimmu.2020.598165 (PMC7883686; doi:10.3389/fimmu.2020.598165)

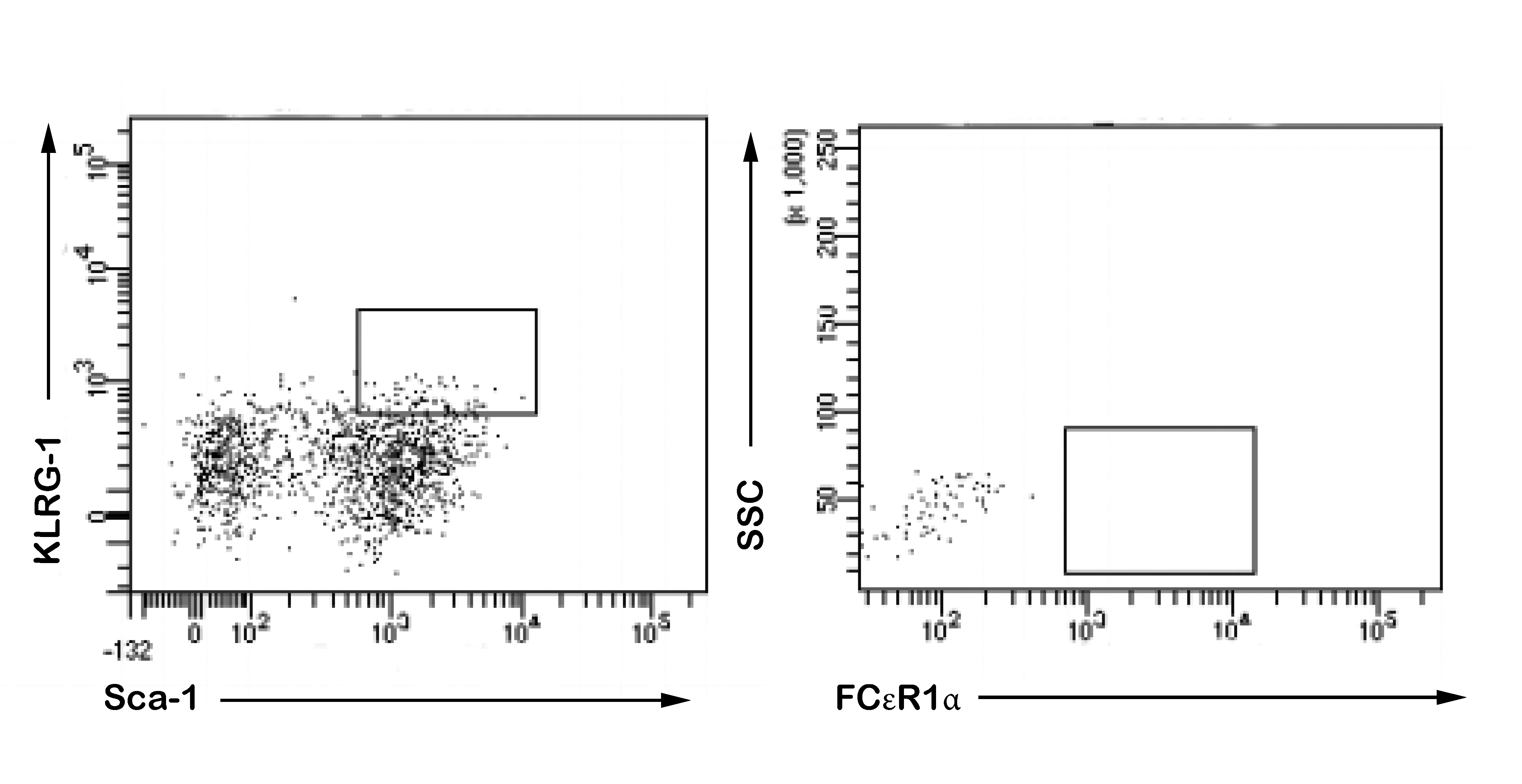

Supplement: Supplementary file 1 [file Image_1.tiff]

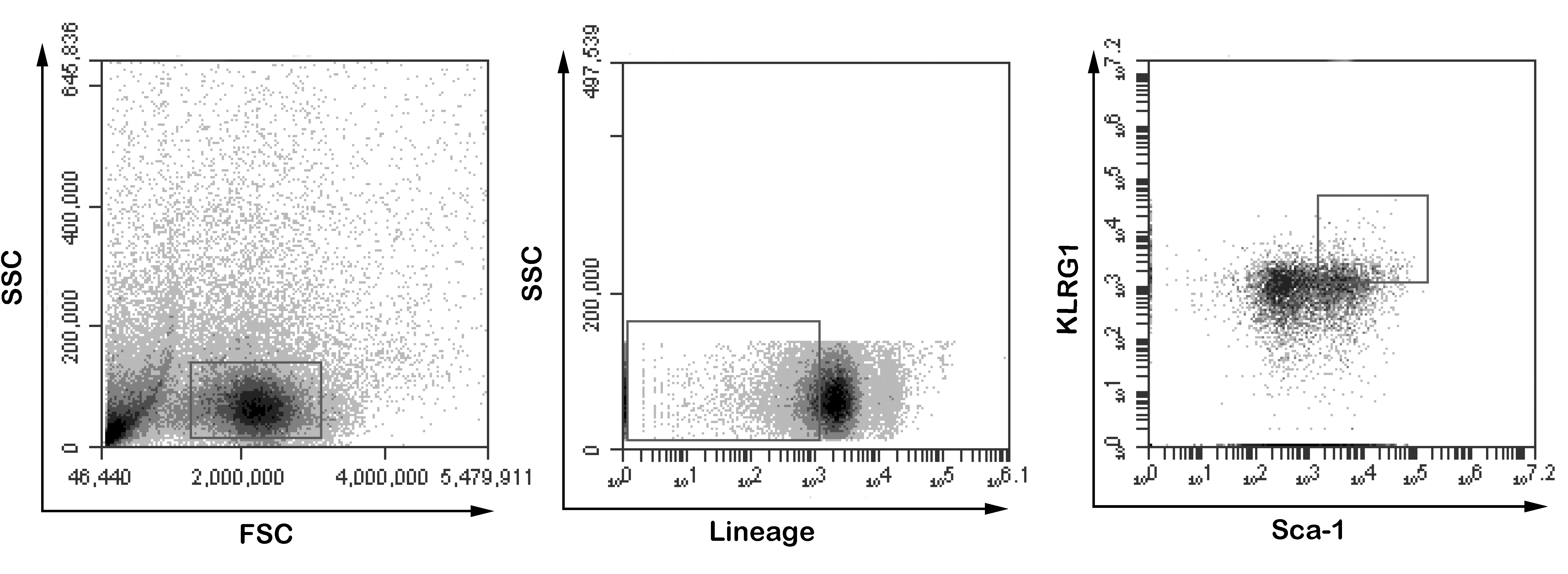

Supplement: Supplementary file 2 [file Image_2.tiff]

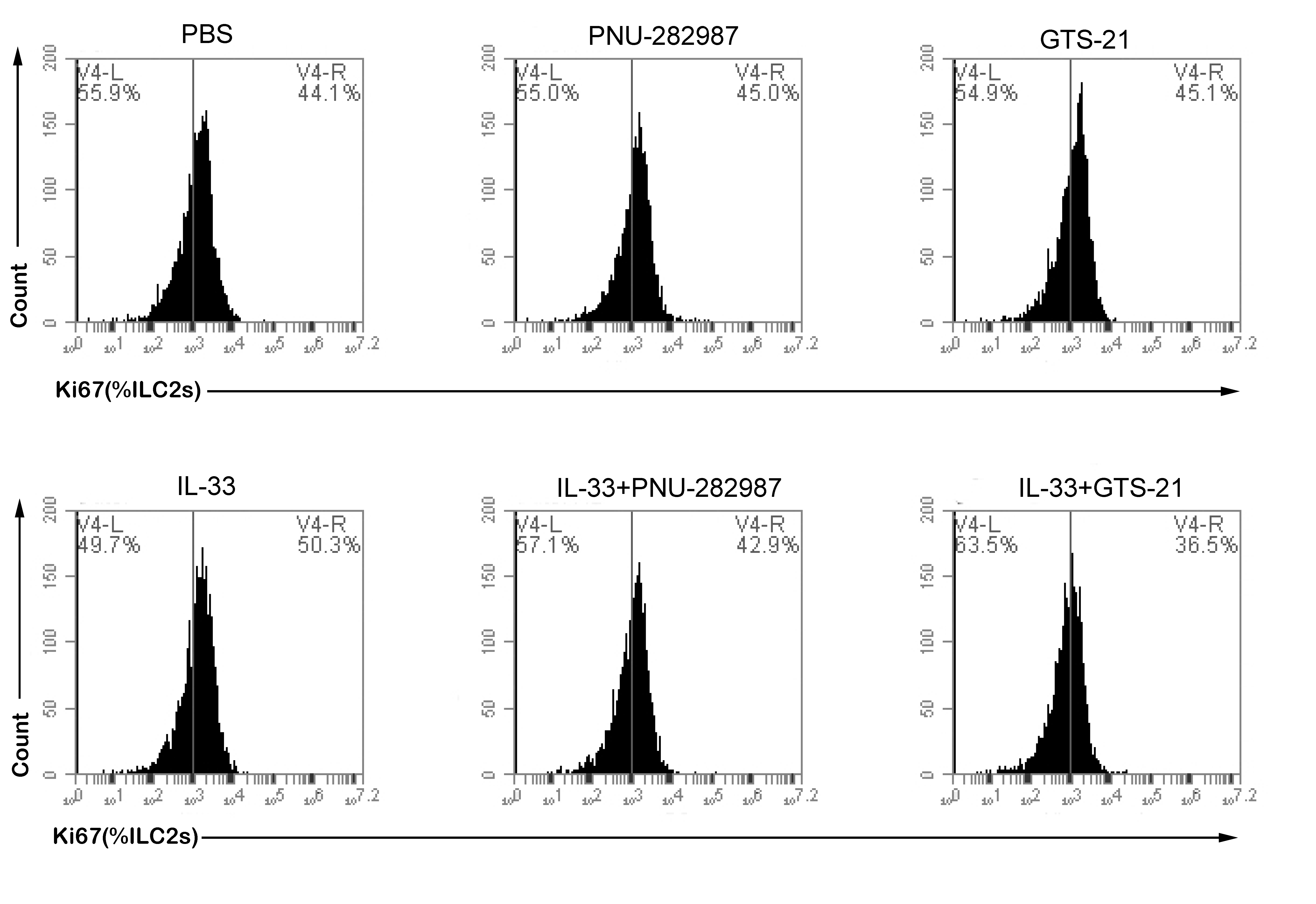

Supplement: Supplementary file 3 [file Image_3.tiff]

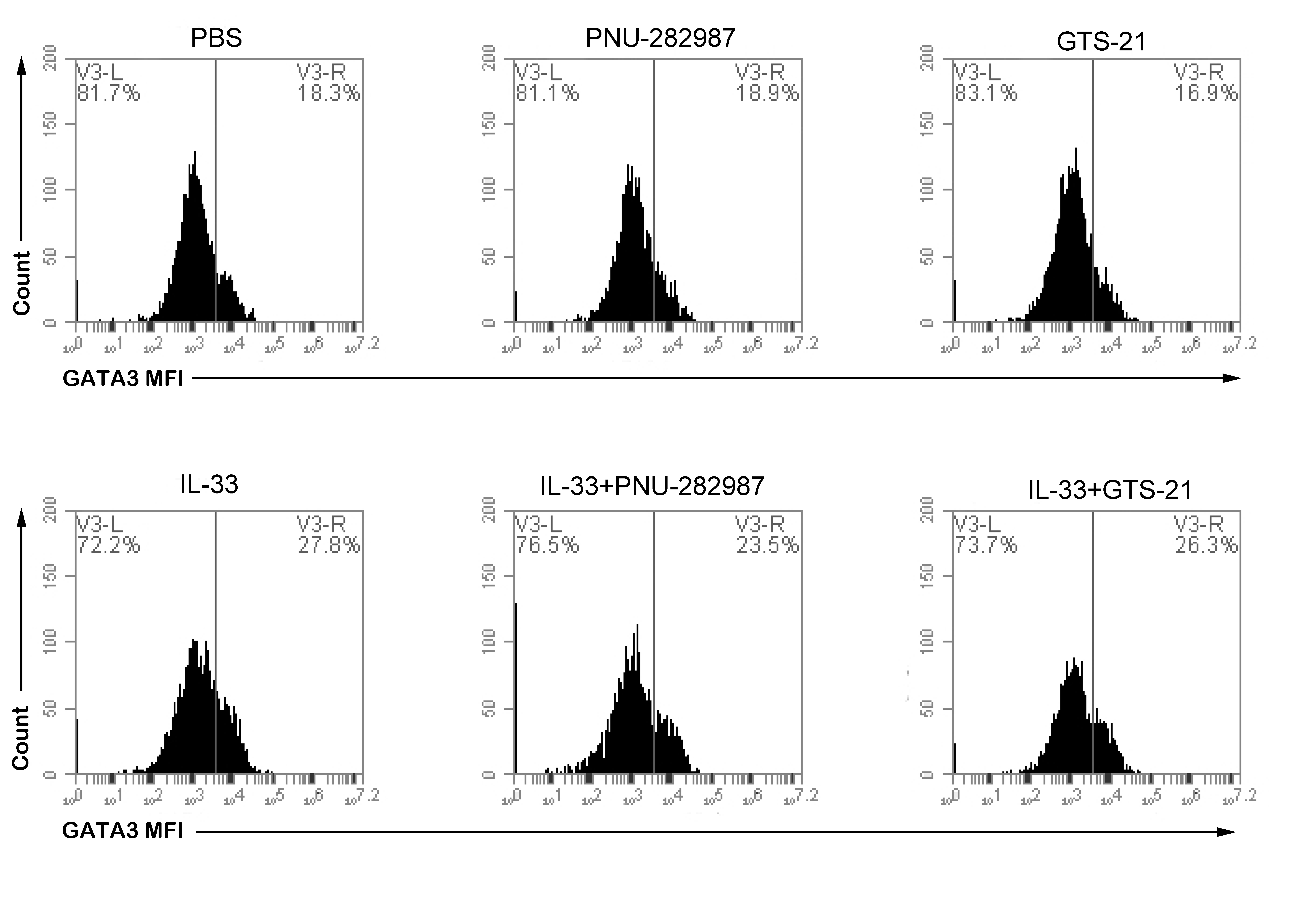

Supplement: Supplementary file 4 [file Image_4.tiff]

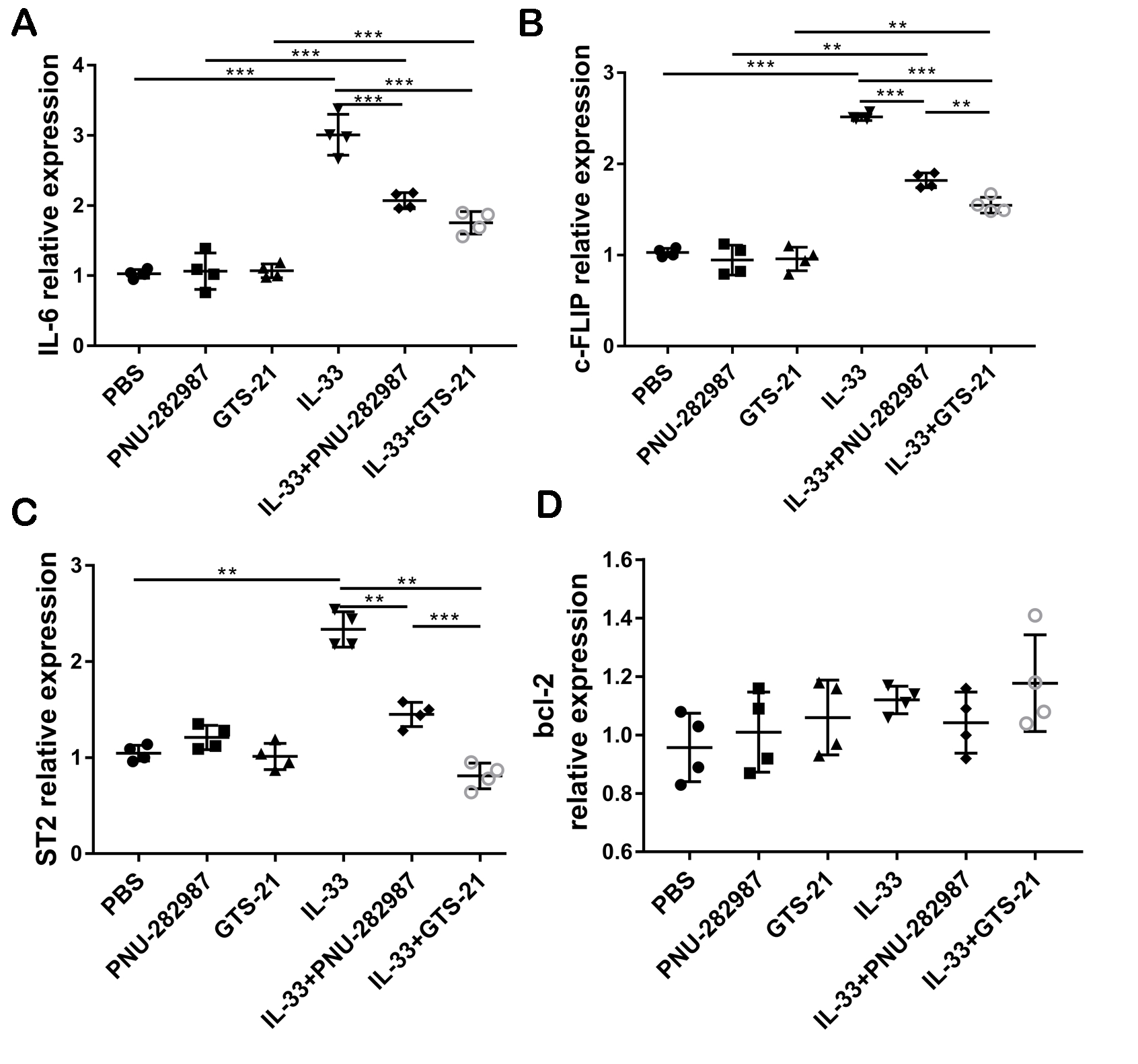

Supplement: Supplementary file 5 [file Image_5.tiff]

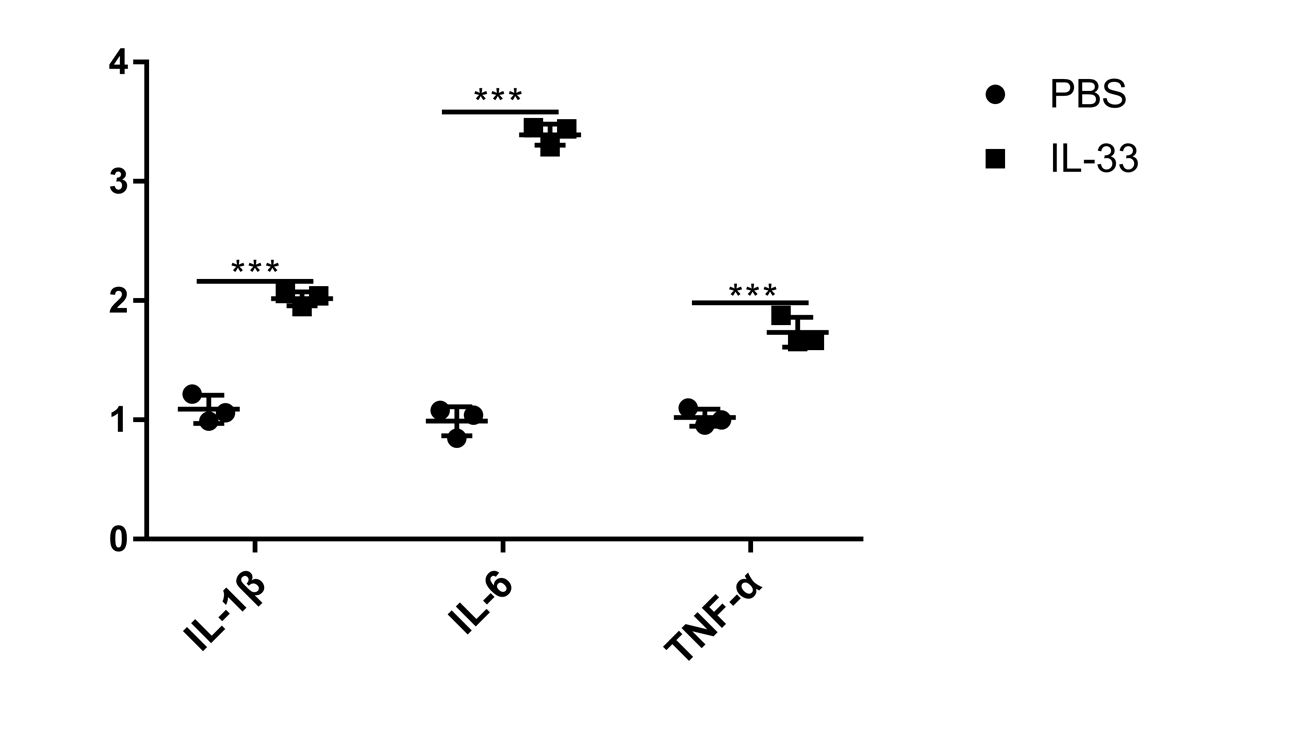

Supplement: Supplementary file 6 [file Image_6.tiff]

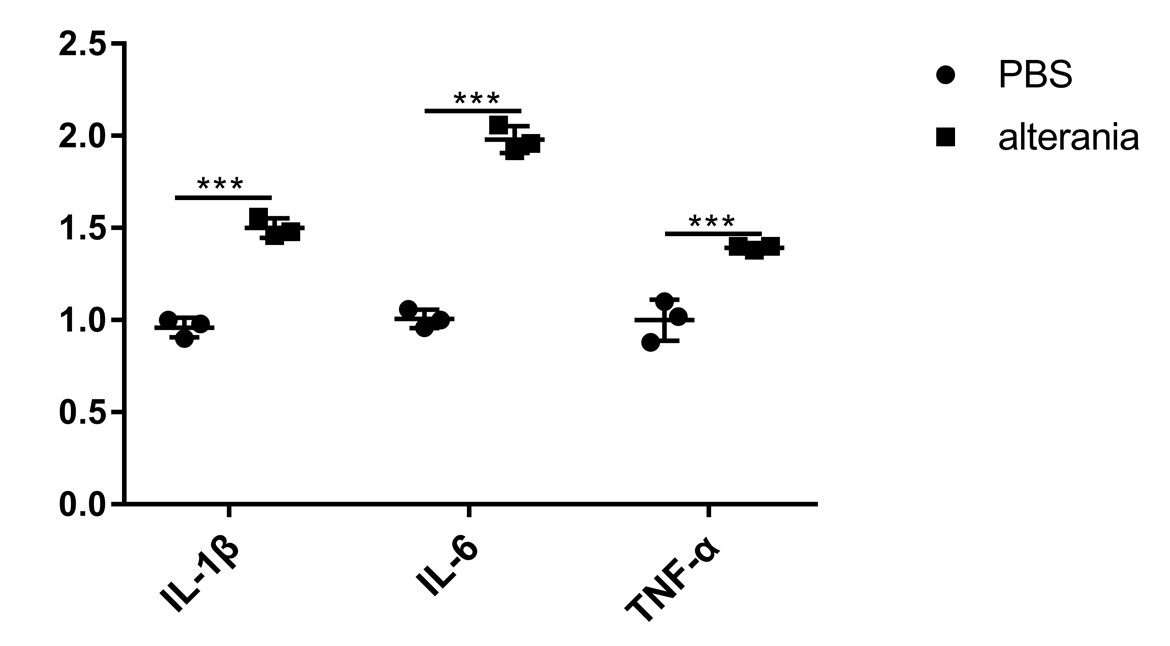

Supplement: Supplementary file 7 [file Image_7.tiff]
