# Supplementary material for: A Selective α7 Nicotinic Acetylcholine Receptor Agonist, PNU-282987, Attenuates ILC2s Activation and Alternaria-Induced Airway Inflammation
Source: Front Immunol. 2021 Feb 1;11:598165. doi: 10.3389/fimmu.2020.598165 (PMC7883686; doi:10.3389/fimmu.2020.598165)
Supplement: Supplementary file 8 [file Table_1.docx]

**Supplementary Figure Legends**

**Fig S1:** C57BL/6J mice intranasally challenged with recombinant mouse IL-33 on days 1 and 3. Percentage of FcƐR1α^+^cells in lung ILC2s (KLRG1^+^Sca-1^+^) were assessed by flow cytometry.

**Fig S2:** Gating strategy for purification of ILC2s by flow cytometry analysis. ILC2s were defined by a lack of lineage markers (CD3e, CD45R, Gr-1, CD11b, CD11c, 7-4 and Ter119, NK1.1, TCR-β), KLRG1^+^ and Sca-1^+^ populations.

**Fig S3:** Representative plots of Ki67^+^cells in isolated lung ILC2s, stimulated with IL-33 in the absence and presence of PNU-282987 and GTS-21.

**Fig S4:** Representative plots of mean fluorescence intensity of GATA3 in isolated lung ILC2s, stimulated with IL-33 in the absence and presence of PNU-282987 and GTS-21.

**Fig S5:** Lin^-^ cells from C57/BL6J female mice were first separated by magnetic beads, and ILC2s were isolated by FACS. The levels of (A) IL-6, (B) c-FLIP, (C) ST2 and (D) bcl-2 from treated ILC2s were measured by qPCR. Data are representative of at least two independent experiments and presented as means ± s.e.m (n=4; ***P*﹤0.01; ****P*﹤0.001).

**Fig S6:** Mice were intranasally challenged with recombinant mouse IL-33 or PBS on days 1 to 3. RNA transcripts of IL-1β, IL-6 and TNF-ɑ of mouse lung tissue were measured by qPCR. Data presented as means ± s.e.m (n=3; ****P*﹤0.001).

**Fig S7:** Mice intranasally challenged with *Alternaria* or PBS on days 1 to 4. RNA transcripts of IL-1β, IL-6 and TNF-ɑ of mouse lung tissue were measured by qPCR. Data presented as means ± s.e.m (n=3; ****P*﹤0.001).
